# A preliminary review of studies on adaptogens: comparison of their bioactivity in TCM with that of ginseng-like herbs used worldwide

**DOI:** 10.1186/s13020-018-0214-9

**Published:** 2018-11-16

**Authors:** Lian-ying Liao, Yi-fan He, Li Li, Hong Meng, Yin-mao Dong, Fan Yi, Pei-gen Xiao

**Affiliations:** 1 Key Laboratory of Cosmetics, China National Light Industry, Beijing Technology and Business University, No. 11/33, Fucheng Road, Haidian District, Beijing, 100048 People’s Republic of China; 2 Beijing Key Laboratory of Plant Resources Research and Development, Beijing Technology and Business University, No. 11/33, Fucheng Road, Haidian District, Beijing, 100048 People’s Republic of China; 3 Institute of Medicinal Plant Development, Chinese Academy of Medical Sciences, Peking Union Medical College, Beijing, 151 Malianwa North Road, Haidian District, Beijing, 100193 People’s Republic of China

**Keywords:** Adaptogen, Immune-neuro-endocrine system, HPA axis, Tonics, Ginseng-like herbs worldwide

## Abstract

Modern studies have shown that adaptogens can non-specifically enhance the resistance of human body under a wide range of external stress conditions with a multi-targeted and multi-channel network-like manner, especially by affect the immune-neuro-endocrine system and the hypothalamic–pituitary–adrenal axis. This review article draws the attention to the relationships of adaptogens, tonics from traditional Chinese medicine (TCM) and ginseng-like herbs worldwide, which all have similar plant sources and clinical applications. To clarify the sources and pharmacological mechanisms of these plant-originated adaptogens, which will provide useful information for the utilization of adaptogens to improve the human health. Meanwhile, the TCMs and the world-wide ginseng-like herbs from each region’s ethnopharmacology will be beneficial modernization and globalization.

## Introduction

The term of adaptogen was first proposed in 1940 by a scientist from the USSR, namely, N. Lazarev, when he described *Schisandra chinensis* (Turcz.) Bail. and other herbs with the following definition: plant-originated adaptogens that can non-specifically the enhance human body. According to the primary definition of adaptogens, these substances must meet three criteria: first, adaptogens must to be non-specific and must assist the human body in resisting a wide range of adverse conditions, such as physical, chemical or biological stress. These may include environmental pollution, climate change, radiation, infectious diseases, and interpersonal disharmony. Second, adaptogens must maintain homeostasis in humans, that is, these substances can offset or resist physical disorders caused by external stress. Third, adaptogens must not harm the normal functions of the human body.

Then I.Brekhman, a Soviet scientist, studied ginseng in approximately 1950, he extended the concept of adaptogens as follows: medicines that have similar functions as adaptogens can help the body maintain ideal homeostasis under adverse or stressful conditions.

Again, Brekhman and Dardymov further defined plant-originated adaptogens in 1969 [1]. According to this definition, plant-originated adaptogens meet four criteria: first, plant-originated adaptogens must reduce the harm caused by stressed states, such as fatigue, infection, and depression; second, plant-originated adaptogens must have positive excitatory effects on the human body; third, in contrast to traditional stimulants, the excitatory effects produced by plant-originated adaptogens must not cause side effects such as insomnia, low protein synthesis, or excessive energy consumption; fourth, plant-originated adaptogens must not harm the human body.

In the 1990s, a group of scientists, comprised of Hildebert Wagner, George Wikman and Alexander Panossian, performed many studies on adaptogens and proposed the following definition: adaptogens are natural bioregulators that increase the ability to adapt environmental factors and avoid the damage caused by those factors. In fact, the advantage of adaptogens are they minimized the bodily response to stress, reducing the negative reactions during the alarm phase and eliminating, or at least decreasing, the onset of the exhaustion phase that is part of the so-called general adaptation syndrome [2].

With continuous research for more than half a century, the concept of adaptogens has been continuously modified and perfected. In 1998, the American Food and Drug Administration (FDA) [3] defined an adaptogen as a new kind of metabolic regulator that has been proved to help in environmental adaptation and to prevent external harms. Adaptogen has been generally used as a functional term.

Yance, an American herbal doctor, held a view that adaptogens can improve our ability to recognize, respond, recover, and restore or regenerate. He divided adaptogens into three categories, including primary adaptogens, secondary adaptogens, and adaptogen companions, based on his clinical experience [4]. Primary adaptogens are consistent with the traditional definition of adaptogens and satisfy specific criterias. The criterias contains: abundant scientific research confirmed their adaptogenic characters, guarantee of general resistance and non-specific action in the human body, maintenance or restoration for homeostasis, and adverse- or toxic-effects after prolonged use [5].

Furthermore, some precise scientific experiments demonstrated that adaptogens can enhance the resistance of human body against various external stimuli as non-specific regulators. Adaptogen function mainly by affecting the hypothalamic–pituitary–adrenal (HPA axis) in response to stimulation by external stress. Primary adaptogens can not only maintain or recover homeostasis and allostasis but can also promote anabolic recovery. Primary adaptogens can produce positive stress response and the associated hormone expression. Primary adaptogens strengthen the functioning of each systems, promote optimal response, promote recovery of function, and help regulate energy use by improving the function of neuroendocrine system and enhancing cellular energy transfer, which can make body utilize oxygen, glucose, lipids and proteins very effectively, thus providing us with a steady supply of energy [4].

Another category of adaptogens is secondary adaptogens, which are consistent with most traditional definitions of adaptogens but not all of the criteria of primary adaptogens. Secondary adaptogens cannot influence the HPA axis directly; however, these adaptogens can affect the immune, nervous and endocrine systems. Secondary adaptogens share several common features: first, these adaptogens typically exert influence on the immune, nervous and endocrine systems; second, these adaptogens do not influence the HPA axis, directly; third, these plant-originated adaptogens include fatty acids, sterols and phenols; fourth, these substances can enhance anabolism. While the secondary adaptogens may meet most of the qualifications of primary adaptogens, but they have yet to be studied extensively [5].

Another category is adaptogen companions, which may not satisfy all the traditional standards but can have beneficial effects on the HPA axis and on anabolism to support the functions of adaptogens. Although these kinds of medicinal plants have similar functions as the other two kinds of adaptogens mentioned above, these plants can not formally be called adaptogen. Thus, these plants are classified as adaptogens companions because they can act synergistically with the other two kinds of adaptogens mentioned above, thereby improving the effects of the adaptogens [5].

Currently, studies have confirmed that the following plants are true adaptogens: *Panax ginseng* C.A.Mey, *Schisandra chinensis* (Turcz.) Baill., *Acanthopanax senticosus* (Rupr. et Maxim.) Harms, *Rhodiola crenulata* (Hook. f. et Thoms) S.H.Fu, and *Lepidium meyenii* Walp.

According to web of science database, we searched the key word, adaptogen. Based on the analysis of results, we got a conclusion that more and more researches focus on this field over past 10 years and it showed an increasing tendency from 1999 to 2018, which profiles that it is still a worthwhile direction to explore. Furthermore, we also found out that the many published articles concentrated on the direction of pharmacology pharmacy, biochemistry molecular biology, plant sciences and agriculture. It is still worthy to show its historical development and find out the relationships among adaptogen and tonics and ginseng-like herbs worldwide, which can give a hint to further research in plant-originated adaptogen. Summarized the results of previous studies and our own researches focused on tonics from TCM, we launched out our understanding of adaptogen: Herbs which can non-specific and non-toxicity help human body resist the environmental stress to maintain a homeostasis. The mechanism may multi-targets and multi-channel network to the neuroendocrine system. The adaptogens with tonics from TCM and the world-wide ginseng-like herbs have similar phytochemical and pharmacological properties.

## The functions of adaptogens

Adaptogens can affect different tissues and organs, and adjust each of these parts to attain homeostasis.

### Adaptogens and adrenal fatigue

Adrenals, the glands of stress, mobilize various stress responses to each stress, including physical, biochemical, hormonal, thermal, internal, external, emotional and mental. Stress rather than pathological damage is the primary cause of adrenal fatigue. Excessive stress may be caused by a single strong stimulatory event or by the accumulation of chronic or repeated stress. When the capacity of the adrenals to secrete enough hormones that can make the necessary physiological, and biochemical compensations for that level of stress cannot meet the requirements of continually excessive pressure, adrenal fatigue occurs. The adrenal gland continues to work under adrenal fatigue but cannot maintain normal homeostasis. On the one hand, if the adrenals can deal well with this circumstance and cortisol levels remain adequately elevated to handle the various stresses, over time, the signs and symptoms of metabolic syndrome, such as muscle wastage, hyperglycemia, and suppresses immune or inflammatory responses [6], begin to appear. On the other hand, if the adrenals are not able to meet the demands, adrenal fatigue appears, usually developing more quickly than metabolic syndrome, and which can become so severe as to disable them [7].

The amount of stress hormone produced by the human body increases under external pressure. Adaptogens can increase the effectiveness of adrenal gland secretion, thereby abolishing excess hormone production [8]. Other studies can prove this statement: In 2001, B.T.Gaffney found that the suggested that Panax ginseng inhibits 11-beta hydroxysteroid dehydrogenase one and *Eleutherococcus senticosus* inhibits catechol-*O*-methyl transferase, both of which reside in close proximity to stress hormone receptors and catalyze the degradation of stress hormones into inactive compounds [9]. In the absence of stress, adaptogens can accelerate the closure of the adrenal gland. Furthermore, adaptogens can increase cellular energy levels and prevent oxidative damages, leading to the maintenance of normal adrenal function. The following plant-originated adaptogens support adrenal function: *Panax quinquefolius* L. [10], *Withania somnifera* [11], *Panax ginseng* C.A.Mey. [9], *Codonopsis pilosula* (Franch.)Nannf. [12], *Eleutherococcus senticosus* (Rupr. & Maxim.) Maxim. [13], *Gynostemma pentaphyllum* (Thunb.) Makino, *Glycyrrhiza uralensis* Fisch.ex DC. [14], *Ganoderma Lucidum* Karst [15], and *Sedum rosea* (L.) Scop [16].

### Adaptogens and arthritis

Arthritis is caused by tissue damages and joint diseases, which is typically accompanied by pain and swelling. The most common types of arthritis are osteoarthritis and rheumatoid arthritis. Fibromyalgia maybe an accompanying condition of arthritis; however, it is not considered a form of arthritis because it does not cause inflammation or joint damage.

Adaptogens can reduce arthritis-associated inflammation and pain effectively [17, 18]. The anti-inflammatory effects of the following plant-originated adaptogens can be used to provide relief rheumatoid arthritis: *Withania somnifera* [8, 19], *Panax ginseng* C.A.Mey [20], *Gynostemma pentaphyllum* (Thunb.) Makino [21], *Ganoderma Lucidum Karst* [22], *Sedum rosea* (L.) Scop. [23], and *Glycyrhiza uralensis* Fisch. ex DC. [24].

### Adaptogens and sleep

Many people suffer from insomnia and other sleep-related problems. External stress perturbs the normal secretion of circadian cortisol, which is the main cause of sleep-related problems. The secretion of cortisol follows the biological clock and external circadian rhythms. The secretion of cortisol peaks in the morning and then decreases, reaching a minimum value at night. Proper exercise, diet and sleep can help maintain stable cortisol levels in the human body.

Adaptogens help produce cortisol and relieve stress [7, 25]. Studies by Steven Maimes and N. V. Provalova suggest that the following plant-originated adaptogens can act as sleep aids: *Panax quniquefolius* L. [26], *Withania somnifera* (L.) Dunal. [27], *Schisandra chinensis* (Turcz.) Baill. [28], *Gynostemma pentaphyllum* (Thunb.) Makino [29], and *Sedum rosea* (L.) Scop [30].

The following plant-originated adaptogens can alleviate the effects of the time difference syndrome, caused by disruption of physiological rhythm of the human body: *Panax quniquefolius* L., *Panax ginseng* C.A.Mey, *Gynostemma pentaphyllum* (Thunb.) Makino, *Schisandra chinensis* (Turcz.) Baill., and *Sedum rosea* (L.) [31, 32].

### Adaptogens and the neuroendocrine system

One of the most important functions of adaptogens is their ability to help stabilize the internal environment of the human body by affecting the neuroendocrine system. The chemicals in plant-originated adaptogens enhance the ability to adapt to external environments and avoid damage [33–35]. A unique feature of adaptogens is that these substances affect the neuroendocrine system and the cellular energy system. Aaptogens can increase the rates of oxygen, protein, fat and sugar utilization. In addition to plant-originated adaptogens, other plants may have some of the functions mentioned above, but plant-originated adaptogens have a broad range of functions and systematically strengthen the stability of the internal environment of the human body [34, 36].

### Anti-tumor application of plant-originated adaptogens

Researchers have found that plant-originated adaptogens have a positive influence on all aspects of the health of animals and humans [37]. Cancers studies have shown that plant-originated adaptogen can reduce the risks of cancers [38].

Plant-originated adaptogens play key roles in anti-tumor and multifaceted anticancer mechanisms, such as inhibition of cancer cells production, stabilization of the functions of human body, and promotion of cell repair [39, 40]. The anti-tumor effect of adaptogens is often closely linked to immune mechanisms. In other words, adaptogens can activate macrophages, T-lymphocytes, NK cells and so on to inhibit the growth of tumors and enhance cell-selective apoptosis and intercellular connection [16, 41–43].

The most common chemical anti-tumor medicines currently on the market have side effects such as cytotoxicity and immune suppression. Plant-based immune regulators, for example, plant-originated adaptogens, are generally used as auxiliary treatments to reduce the side effects of these chemical medicines and regain health [44, 45]. Notably, the application of adaptogens improves the tolerance of humans to drug cytotoxicity [46]. Adaptogens can improve the physical conditions of cancer patients in the following ways: first, as modulators of biological responses, adaptogens can the remodel immune mechanism and non-specifically enhance the resistance of the human body [46]; second, adaptogens can promote the production of marrow, increase the amount of blood cells and reduce infection [47]; third, adaptogens affect the entire body, from cells to organs including the liver, kidney, heart and gastrointestinal tract [7]; fourth, adaptogens can strengthen the lethal effects of chemotherapy and radiotherapy on cancer cells [48]; fifth, adaptogens can inhibit the development of multidrug resistance [49]; sixth, adaptogens can inhibit tumor metastasis and cancer cell aggregation [50]; seven, adaptogens can reduce stress hormone levels during immune dysfunction, which is associated with tumor growth [51].

When cancer cells adapt to chemotherapy, the main consequence is the development of multidrug resistance. The most simple mechanism of the development of multidrug resistance is as follows: anti-tumor drug molecules flow out from cancer cells through membrane channel proteins driven by ATP, especially by adjustment of P-glycoprotein pump (Pgp-pump) and due to the effects of breast cancer resistance protein-1 (BCRP/ABCG-2) and multidrug-resistance-associated protein-1 (MRP-1) from thymic cancer cells [52]. Among plant-originated adaptogens, *Panax ginseng* C.A.Mey can substantially reduce multidrug resistance by inhibiting Pgp, and it has been shown that *Panax ginseng* C.A.Mey can prolong the life of the cancer-bearing mice in animal experiments [24, 53]. In addition, epigallocatechin-gallate (EGCG) in *Eurycoma longifolia* can inhibit the expression of Bcl-2 to prevent the development multidrug resistance [54].

## Exploration of the mechanism of action of adaptogens

**Fig. 1:**
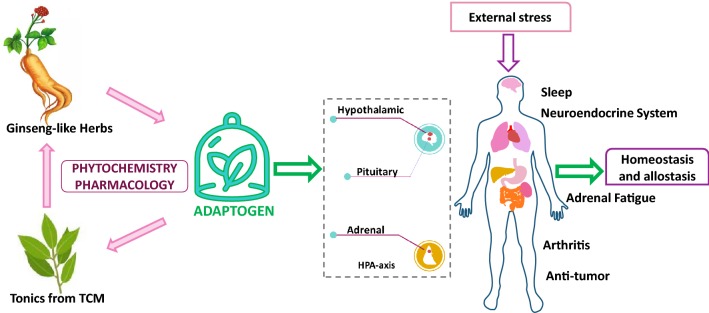
The mechanism of action of plant-originated adaptogens on human body

In the case of various stress modes, adaptogens can activate the adjustment of different responses to cope with different forms of stress. Adaptogens are the material basis of the bodily response to the external environment and can act on the immune system and the stress response system, showing in Fig. 1. The non-specific response mode, especially the hormone response mode, occurs when homeostasis is not the driving force. When hormone levels exceed the critical level, the complex neurosecretory reactions may have harmful effects [55, 56]. The human stress response system consists of the central nervous system (CNS), which includes neurons of the hypothalamic paraventricular nucleus, and is associated with to corticotropin releasing hormone (CRH), and arginine vasopressin (AVP), and the adrenalin nucleus as well as its distal ends of the brainstem, the HPA axis and the peripheral nervous system [57]. The central coordination system to respond to external pressure consists of CRH neurons, AVP neurons, catecholamine neurons and other cell tissues, and the HPA axis and sympathetic nervous system (SNS) represent the limbs [58]. CRH and catecholamine neurons interact with each other. The SNS and HPA system interact in terms of functions and systematic anatomy. When responding to the external environment, these systems can interact on different levels, for example, catecholamine can stimulate the HPA axis by releasing of CRH, and the hormone produced by the HPA axis can act on the SNS system [33].

Recent studies have shown that the inhibitory effects and long-term overexpression of endogenous glucocorticoids cause stimulatory effects that are adjusted by SNS under stress. The secretion of CRH and AVP increases, which is stimulated by external pressure, thereby promoting the secretion of cortisol and adrenocorticotropic hormone. Furthermore, angiotensin, cytokines and arachidonic acid metabolites participate in the stress response. SNS provides the human body with a fast response mechanism to external stress. In addition to catecholamine, the sympathetic and parasympathetic nervous systems can also secrete a variety of neuropeptides, ATP and nitric oxide (NO) [59]. The following effects are observed upon adjustment the HPA axis: an increase in and regulation of energy circulation; reduction in the feeling of external pressure; enhancement of resistance; improvement of mental concentration; facilitation of deep sleep period after sleep. These functions are all considered the primary function of adaptogens [55, 56, 60].

Adaptogens do not increase the levels of cortisol and NO in the human body under acute physiological loads [6, 31, 61]. Plant-originated adaptogens, for example, *Schisandra chinensis* (Turcz.) Baill, can prevent and resist stress because these substances can activate the secretion of cortisol and NO in the plasms and saliva, allowing the body to adapt to heavier loads. After the consumption of plant-originated adaptogens, physical exercises do not increase the level of cortisol and NO in the human body; in fact, the levels decrease, comparing to those present prior to physical exercise. Thus, adaptogens can increase the level of messenger substances that activate stress (NO) and suppress stress (cortisol).

Adaptogens can improve the stress response system to respond to high levels of external signals in the normal or abnormal states. We should determine the similarities or differences between adaptogens and known classical metabolic regulators. According to the above description, the main difference may be that adaptogen can stimulate the CNS. It is now possible to obtain additional information at the biochemical level and identify analogues adrenal cortex hormone analogue, catecholamine analogue and so indicating that the active ingredients have similar structures.

## Adaptogens and Chinese tonics

There is much overlap between these medicines and many tonics are internationally recognized as plant-originated adaptogens [2].

In traditional Chinese medicine, it is believed that harmony and balance are indispensable for health and the concepts of yin and yang are used to diagnose and cure disease [62]. Medicines that can supplement deficiency and weakness, correct the pathological bias caused by deficiency in both qi and blood, and treat the syndrome of qi deficiency are called tonics in traditional Chinese medicine (Qì, in the context of Chinese medicine, can be defined as the physiological processes of the body.). According to the differences in the properties, functions and indications of tonics, these medicines can be divided into qi-supplementing medicines, yang-supplementing medicines, blood-supplementing medicines, and yin-supplementing medicines. The common species of tonics and their functions are listed in Table 1.

**Table 1:** Various tonics and their features

Classification	Scientific name	Immunity	Neuroendocrine	Anti-stress	Anti-inflammatory	Anti-age	Hypolipidemic	Hypoglycemic	Anti-tumor	References
Qi-supplementing medicines	*Panax ginseng* C.A.Mey	√	√	√	√	√	√	√	√	[42]
	*Codonopsis pilosula* (Franch.) Nannf.	√	√	√	√	√		√	√	[63]
	*Pannax quinquefolius* L.	√	√	√			√			[42]
	*Rhodiola crenulata* (Hook.f. et Thoms) S.H.Fu	√	√	√	√	√				[48]
	*Astragalus membranaceus* (Fisch.) Bunge.	√	√	√	√	√				[64]
	*Glycyrrhiza uralensis* Fisch.	√	√		√		√			[65]
	*Acanthopan*-*ax senticosus* (Rupr. et Maxim.) Harms	√	√	√	√			√	√	[13]
	*Atractylodes macrocephala* Koidz.	√	√	√					√	[64]
Blood-supplementing medicines	*Angelica sinensis* (Oliv.) Diels	√			√	√	√			[64]
	*Fallopia multiflora* (Thunb.) Harald.	√				√	√			[66]
	*Cynanchum otophyllum* Schneid.	√	√		√					[67]
Yang-supplementing medicines	*Cuscuta chinensis* Lam.	√	√							[68]
	*Epimedium brevicornu* Maxim.	√	√	√	√					[69]
	*Psoralea corylifolia* Linn.	√	√						√	[70]
	*Stachys geobombycis* C.Y.Wu	√	√	√		√			√	[68]
	Cynomorium songaricum *Rupr.*	√	√							[71]
Yin-supplementing medicines	*Asparagus cochinchinensis* (Lour.) Merr.	√		√	√				√	[72]
	*Ophiopogon japonicus* (Linn. f.) Ker-Gawl	√	√		√	√		√		[73]
	*Polygonatum odoratum*	√	√		√					[74]
	*Fructus Ligustri* Lucidi.	√		√						[75]
	*Cornus officinalis* Sieb.et Zucc.	√				√				[76]
	*Lycium chinense* Mill.	√		√		√	√	√	√	[77]

Tonics have wide range of applications in traditional Chinese medicine and can be used under the conditions of low body resistance and weak constitution or when the human body is finding it difficult to fight severe diseases, this function of tonics is similar to that of plant-originated adaptogens [78]. Tonics typically are adaptogens, but not always. They are similar in the following aspects: First, the clinical application and mechanisms of action of these substances are similar. Based on their current applications, tonics and plant-originated adaptogens can be described as substances that regulate human bodily functions in order to attain homeostasis. The primary effects exhibited by these substances are regulation of the immune system, improving the disorders of the nervous system, anti-fatigue effects and overall nourishment. In terms of specific mechanisms, both medicines can affect the HPA axis in the immune-neuro-endocrine system, thereby achieving the above pharmacological effects [79]. Second, the plants of origin of these substances are similar [2]. Chinese herbs considered to be both adaptogens and tonics include the following: *Panax ginseng* C.A.Mey, *Panax quniquefolius* L., *Panax notoginseng* (Burkill) F.H.Chen, *Eleutherococcus senticosus* (Rupr. & Maxim.) Maxim, *Sedum rosea* (L.) Scop., and *Schisandra chinensis* (Turcz.) Baill. According to the terminology used in traditional Chinese medicine, the mechanism of action of plant-originated adaptogens is to achieve equilibrium in both yin and yang, showing great vitality [80].

Based on the chemical composition, common tonics can be divided into following several categories in Table 2.

**Table 2:** Based on the chemical composition, common tonics can be divided into following several categories

Category	Specific tonics
Saponin	*Panax ginseng* C.A.Mey.
	*Panax japonicus* C.A.Mey.
	*Codonopsis pilosula* (Franch.) Nannf.
	*Codonopsis lanceolata* (Siebold & Zucc.) Benth. & Hook.f. ex Trautv.
	*Glycyrrhiza uralensis* Fisch. ex DC.
	*Eleutherococcus senticosus* (Rupr. & Maxim.) Maxim.
	*Ophiopogon japonicus* (Thunb.) Ker Gawl.
	*Asparagus cochinchinensis* (Lour.) Merr.
	*Cornus officinalis* Siebold & Zucc.
	*Trigonella foenum*-*graecum* L.
	*Ipomoea batatas* (L.) Lam.
	*Ziziphus jujuba* Mill.
	*Broussonetia papyrifera* (L.) L’Hér. ex Vent.
	*Cynomorium songaricum* Rupr.
	*Ligustrum lucidum* W.T.Aiton
Coumarin	*Psoralea corylifolia* L.
	*Clinopodium megalanthum* (Diels) C.Y.Wu & S.J.Hsuan ex H.W.Li
	*Lycium chinense* Mill.
	*Glycyrrhiza uralensis* Fisch. ex DC.
	*Eleutherococcus senticosus* (Rupr. & Maxim.) Maxim.
	*Panax ginseng* C.A.Mey.
	*Astragalus membranaceus* Fisch. ex Bunge
Flavonoids	*Epimedium brevicornu* Maxim.
	*Astragalus membranaceus* Fisch. ex Bunge
	*Panax ginseng* C.A.Mey.
	*Eucommia ulmoides* Oliver
	*Psoralea corylifolia* L.
	*Glycyrrhiza uralensis* Fisch. ex DC.
	*Cuscuta chinensis* Lam.
	*Trigonella foenum*-*graecum* L.
	*Taxillus sutchuenensis* (Lecomte) Danser
	*Dendrobium nobile* Lindl.
Alkaloids	*Trigonella foenum*-*graecum* L.
	*Epimedium brevicornu* Maxim.
	*Cistanche deserticola* Ma
	*Polygonatum odoratum* (Mill.) Druce
	*Phlomis umbrosa* Turczaninow
	*Ilex cornuta* Lindl. & Paxton
	*Allium tuberosum* Rottler ex Spreng.
	*Cuscuta chinensis* Lam.
	*Dendrobium nobile* Lindl.
	*Aconitum carmichaeli* Debeaux
	*Lilium brownii* var.viridulum Baker
Volatile oils	*Atractylodes macrocephala* Koidz.
	*Alpinia oxyphylla* Miq.
	*Angelica sinensis* (Oliv.) Diels
	*Clinopodium megalanthum* (Diels) C.Y. Wu & Hsuan ex H.W.
	*Psoralea corylifolia* L.
	*Epimedium brevicornu* Maxim.
	*Trigonella foenum*-*graecum* L.
Amino acids	*Rehmannia glutinosa* (Gaertn.) DC.
	*Stachys geobombycis* C.Y.Wu
	*Asparagus cochinchinensis* (Lour.) Merr.
	*Glycyrrhiza uralensis* Fisch. ex DC.
	*Panax ginseng* C.A.Mey
	*Trigonella foenum*-*graecum* L.
	*Lycium chinense* Mill.
Polysaccharides	*Astragalus membranaceus* Fisch. ex Bunge
	Eleutherococcus senticosus (Rupr. & Maxim.) Maxim.
	*Polygonatum sibiricum* Redouté
	*Panax ginseng* C.A.Mey.
Other	*Fallopia multiflora* (Thunb.) Haraldson
	*Cynanchum otophyllum* C.K.Schneid.
	*Rehmannia glutinosa* (Gaertn.) DC.
	*Epimedium brevicornu* Maxim.
	*Eucommia ulmoides* Oliver

According to Panossian’s conclusion, the main active chemical components can be divided into the following two categories. The first category includes terpenoids with four-ring skeletons that are similar to cortisol: sitoindosides (*Withania somnifera*), cucurbitacin-R-saponin (*Bryonia dioica*). The second category includes aromatic compounds with structures similar to that of catecholamine: (a) lignans: eleuteroside E (*Acanthopanax senticosus* (Rupr. et Maxim) Harms), schisandrin b (*Schisandra chinensis* (Turcz.)); (b) phenylpropane derivatives: syringin (*Acanthopanax senticosus* (Rupr. et Maxim) Harms), cinnamyl glycoside (*Rhodiola crenulata* (Hook.f. et Thoms) S.H.Fu); (c) phenylethane derivatives: salidroside.

Tonics have the following functions: enriching and activating blood (promoting the secretion of erythropoietin and inducing the production of stimulatory factors, for example, macrophage colonies, to activate blood), regulating cellular and humoral immunity, and affecting cytokine activity. These functions are similar with those of the plant-originated adaptogens mentioned above. Plant-originated adaptogens are generally considered to be the “elite of herbs” [5], and in Chinese medicine, tonics are considered the highest grade of medicine [81]. In traditional Chinese medicine theory, tonics are also considered to be top grades in traditional Chinese medicine. It is helpful to expand the range of plant-originated adaptogen species and to have an in-depth analysis about the mechanism of tonic traditional Chinese medicine.

## Adaptogens and ginseng species worldwide

Many regions, ethnic groups and countries, they have their own medical histories and habits. Continuous development and transfer of knowledge through generations leads to the formation of unique medicinal systems such as traditional Chinese medicine and Indian ayurvedic medicine. Coincidentally, in different regions and medical systems, there are several medicinal plants that are considered to be national treasures or that are called ginseng locally. There is much overlap between plant-originated adaptogens and ginseng species worldwide. Furthermore, most ginseng species worldwide are internationally recognized as plant-originated adaptogens. Both ginseng-like herbs worldwide and plant-originated adaptogens have very similar clinical applications. Ginseng species are widely used by local communities world-wide because these plants can enhance the resistance of the human body and can have various beneficial effects, such as anti-fatigue, anti-ageing, anti-stress, anti-anxiety, anti-inflammatory, and anti-depression. Furthermore, ginseng species may improve the circulatory system and immune system [34], which correlates precisely to the function of adaptogens.

Most medicinal plants called ginseng belong to Araliaceous, but there are also other medicinal plants that belong to other families. For example, *Withania somnifera*, which belongs to Slanaceae, is called Indian ginseng; this plant has nourishing and strengthening effects and can delay ageing [47]. *Panax japonicus*, which belongs to Solanaceae is called Japanese ginseng; this plants have tonifying, strengthening and anti-fatigue effects [82]. *Eurycoma longifolia*, which belongs to Simaroubaceae is called Malaysian ginseng; this plant can be used as a postpartum tonic or aphrodisiac [83]. *Lepidium meyerii*, which belongs to Brassicaceae, is called Peruvian ginseng; this plant can be used for natural nutrient, can enhance fertility effectively, and has anti-fatigue effects. The common ginsengs species from different countries and their functions are listed in The results of modern pharmacological studies show that these medicinal plants called ginseng have effects on the neuroendocrine and immune systems, which is similar to the mechanism of action of plant-originated adaptogens, the elite of herbs. However, there have been few studies on the chemical compositions, mechanisms of action, traditional curative effects and similarities of these types of medicinal plants. Comparisons with ginseng species from around the world are helpful for widening the spectrum of plant-originated adaptogen species and for in-depth analysis of the mechanism of action of ginseng species worldwide. The functions and the main functional ingredients of common ginseng-like herbs worldwide were showed in Table 3.

**Table 3:** Ginseng-like herbs worldwide with similar functions as those of plant-originated adaptogens

Scientific name	Common name	Family	Common function	Main functional ingredient
*Panax ginseng* C.A.Mey	Asian ginseng	Araliaceae	Used for strengthening, nourishing, adjusting blood pressure and restoring heart function, neurasthenia and physical weakness [42]	Ginsenoside [84]
*Pannax quinquefolius* L.	American ginseng	Araliaceae	Used to treat qi and yin deficiency, endogenous heat, kechuan, sputum, asthenic fever, dysphoria, thirst, and dry mouth and throat [42]	Ginsenoside, etc. [84]
*Panax notoginseng* *(Burk.) F.H.Chen*	Notoginseng	Araliaceae	Used for dispersing blood stasis and haemostasis and relieving swelling and pain [85]	Notoginseng saponins, Dencichine, etc. [84]
*Panax japonicus*	Japanese ginseng	Araliaceae	Used for dispersing blood stasis and haemostasis, relieving swelling and pain, eliminating phlegm and arresting coughing and for tonifying deficiency and strengthening [86]	Chikusetsusaponin, pseudo ginsenoside F11, Panax japonicus polysaccharide, etc. [86]
*Panax pseudoginseng* Wall. var. major (Burkill) H. L. Li	Himalayan ginseng	Araliaceae	Used to treat qi and yin deficiency, dysphoria, thirst, asthenia coughing, injuries from falling down, joint pain, haemoptysis, haematemesis and bleeding wounds [86]	Majoroside, etc. [86]
*Eleutherococcus senticosus*	Siberian ginseng	Araliaceae	Used for regulating body disorders, improving the circulatory system, to treat fatigue, for promoting blood circulation for removing blood stasis, invigorating the stomach and for diuresis and so on [13, 87]	Eleutheroside, acanthopanax senticosus polysaccharide, etc. [13, 87]
*Withania somnifera*	Indian ginseng	Solanaceae	Used for strengthening; nourishing;its anti-ageing, anti-stress, antioxidation, anti-tumor, anti-anxiety, anti-inflammatory, anti-depression properties; immune regulation; improving cognitive function and so on [19]	Withanolides and other steroid esters and withanine and other alkaloids [88]
*Pfaffia paniculata*	Brazilian ginseng	Amaranthaceae	Often used as a tonic, to relieve fatigue effectively, improve sexual function, and lower blood pressure, blood sugar and blood lipid levels [89]	Pfaffic acid and its glycoside, for example, pfaffosides A–F, and nortiperpene and its glycoside, etc. [86]
*Eurycoma longifolia*	Malaysian ginseng	Simaroubaceae	Used for its anticancer, anti-malaria properties, improving male sexual dysfunction, sterilizing, lowering blood pressure and treating diabetes [54]	Diterpenes with quassinoid skeletons and alkaloids such as canthin-6-one, mainly, biphenyl lignin and squalene derivatives [54]
*Lepidium meyerii*	Peruvian ginseng	Brassicaceae	Used as s natural nutrient, for enhancing fertility effectively and for its anti-fatigue properties [90]	Marca amide, marca alkaloids, etc. [91]
*Talinum paniculatum*	Folk ginseng	Talinaceae	Used as a medicinal tonic and for strengthening, nourishing, improving qi, replenishing blood, aiding digestion, promoting fluid production, quenching thirst and treating cough and phlegm containing blood [92]	Terpenoids, coumarins, volatile oils, and polysaccharides, etc. [93]

## Current difficulties and prospects of adaptogens

Plant-originated adaptogens have been demonstrated to regulate stress-related changes, at least in animal experiments. However, even after more than 40-years of herbal research, there are very few drugs that have been successfully introduced as adaptogens in modern medicine. Most of these kinds of plant-based medicines are considered to be plant-originated adaptogens and the remaining few are immune enhancers, anabolic agents and antioxidants, which are the same as plant-originated adaptogens. Therefore, there are many difficulties associated with judging whether a plant is a plant-originated adaptogen or not [94].

Adaptogens are stress response modifiers that non-specifically increase resistance to various stressors, thereby promoting adaptation and survival. Adapting to environmental challenges are multistep processes that involve diverse mechanisms and interactions. Multiple molecular networks are involved that coordinate both intracellular and extracellular stress signaling. The metabolic regulation of homeostasis by adaptogens at the cellular and systems levels is associated with multiple targets [95]. To date, the main problem in the research of mechanism of adaptogens, is the lack of suitable stress-response animal models. The stress responses system can be divided into three parts: stressor, stress response and stress performance [96]. The stressor is the object that induces physical strain; therefore, a stressor may be biological (infection), physical (external force, extreme environment), chemical (medicine, ethanol), or psychological (sadness, argument). When an organism is exposed to a stressor, the neuroendocrine system of the body changes. It is easy to study stress performances because these effects are visible [97].

Therefore, stress is known to lead to high blood pressure, myocardial ischemia, depression and even cancer. Briefly, stress performance is the manifestation of the final effect of the stress response on the target organ. However, it remains challenging to study various steps of this stress response [98]. First, this difficulty is associated with the stressor because it is difficult to quantify external stressors, especially psychological stressors, for example, sadness. In addition, we know little about the interactions between stressors and the body. The specific response of mechanisms induced by stressors affect broad-spectrum stress response. Second, this difficulty is associated with the stress response. In the terms of development, hormones, neurotransmitters, neuro-regulators and cytokines, which are involved in the response have been identified, but the specific functions of these proteins divisions remain unknown. The identity of the first substance to regulate the stress response remains unclear, and it has not been determined whether there are different mechanisms for different stressors. There are no definitive results regarding the specific mechanisms and pathway changes of that lead to conversion of the status from unsuitable to suitable. As a result, it is impossible to differentiate and characterize the different stages of stress response (including adaptation), making the response difficult to quantify [97]. Therefore, scientists use “prevention of stress manifestation” as a guideline to evaluate the anti-stress properties of a drug [99]. Third, the difficulty is associated with stress expression. According to the strength and severity of the stressor, different changes are observed. Genetic or other external factors (species, day–night cycles, gender, age and physiological state of tissues or organs) also affect the extent of overall stress expression. The difficulties mentioned above will appear in the process of the mechanism of action of adaptogens; therefore, it is necessary to further discuss the mechanisms, targets, similarities and differences associated with the pharmacological functions.

To date, various studies and practical applications have shown that plant-originated adaptogens are a kind of elite herbal medicine, playing an important role in human health and helping the human body resist various stress factors. However, the clinical application of plant-originated adaptogens and their use in health care products remains in the preliminary stage. Categorization of plant-originated adaptogens, clarification of their pharmacological functions, and the determination of the similarities and differences between adaptogens, tonics and ginseng species worldwide, will help in effective utilization of plant-originated adaptogens, and provide a new way to guarantee human health.
